# Riluzole exerts distinct antitumor effects from a metabotropic glutamate receptor 1-specific inhibitor on breast cancer cells

**DOI:** 10.18632/oncotarget.17961

**Published:** 2017-05-18

**Authors:** Sonia C Dolfi, Daniel J Medina, Aparna Kareddula, Bhavna Paratala, Ashley Rose, Jatinder Dhami, Suzie Chen, Shridar Ganesan, Gillian Mackay, Alexei Vazquez, Kim M Hirshfield

**Affiliations:** ^1^ Department of Medicine, Rutgers Cancer Institute of New Jersey, Rutgers Robert Wood Johnson Medical School, New Brunswick, New Jersey 08901, USA; ^2^ Susan Lehman Cullman Laboratory for Cancer Research, Department of Chemical Biology, Ernest Mario School of Pharmacy, Rutgers, The State University of New Jersey, Piscataway, New Jersey 08854, USA; ^3^ CRUK Beatson Institute, Garscube Estate, Bearsden, Glasgow G61 1BD, UK

**Keywords:** riluzole, breast cancer, glutamate signaling, cell cycle, BAY 36-7620

## Abstract

Recent evidence suggests that glutamate signaling plays an important role in cancer. Riluzole is a glutamate release inhibitor and FDA-approved drug for the treatment of amyotrophic lateral sclerosis. It has been investigated as an inhibitor of cancer cell growth and tumorigenesis with the intention of repurposing it for the treatment of cancer. Riluzole is thought to act by indirectly inhibiting glutamate signaling. However, the specific effects of riluzole in breast cancer cells are not well understood. In this study, the anti-cancer effects of riluzole were explored in a panel of breast cancer cell lines in comparison to the metabotropic glutamate receptor 1-specific inhibitor BAY 36-7620. While both drugs inhibited breast cancer cell proliferation, there were distinct functional effects suggesting that riluzole action may be metabotropic glutamate receptor 1-independent. Riluzole induced mitotic arrest independent of oxidative stress while BAY 36-7620 had no measurable effect on mitosis. BAY 36-7620 had a more pronounced and significant effect on DNA damage than riluzole. Riluzole altered cellular metabolism as demonstrated by changes in oxidative phosphorylation and cellular metabolite levels. These results provide a better understanding of the functional action of riluzole in the treatment of breast cancer.

## INTRODUCTION

Although glutamate signaling was initially characterized in central nervous system (CNS) development and synaptic transmission, its importance in tumorigenesis in multiple organs is becoming better defined [1–6]. Breast, melanoma, and prostate cancer cell lines and human prostate tumors have been shown to release glutamate [7, 8] as well as express glutamate receptors [9]. Efforts have focused on targeting ionotropic and metabotropic glutamate receptors and their downstream pathways as an anti-cancer therapeutic strategy. Ionotropic glutamate receptors are ligand-gated ion channels and contain four classes of receptors (AMPA, NMDA, kainate, and delta receptors). Metabotropic glutamate receptors, a family of seven-transmembrane G protein-coupled receptors, are divided into three groups based on sequence homology and functional similarities. Metabotropic glutamate receptor 1 (GRM1), a member of group I, regulates synaptic signaling in the CNS. At least four GRM1 variants (mGluR1a, b, d, and g) have been detected due to alternative splicing of the gene transcript, and they encode for proteins with 1195, 906, 908, and 887 amino acids, respectively. All variants share the first 886 N-terminal amino acids but have distinct C-terminal regions.

Though GRM1 was initially functionally characterized in CNS biology, its importance in tumorigenesis in organ systems outside of the CNS has become better defined. GRM1 was first recognized for its oncogenic potential after a transgenic mouse model presented with spontaneous melanomas [10]. This resulted because of a transgene insertion into intron 3 of GRM1 leading to ectopic GRM1 expression. Activation of GRM1 by its natural ligand glutamate or glutamate receptor agonists alters its interactions with G proteins. These interactions lead to stimulation of second messengers and induction of protein kinase C (PKC) and phospholipase C (PLC) and intracellular calcium release with resultant activation of mitogen-activated protein kinase (MAPK) and Akt pathways.

As the drug riluzole blocks glutamate release [11, 12], it indirectly affects glutamate signaling by both autocrine and paracrine actions. *In vitro* data with melanoma cells suggest that riluzole causes increased intracellular glutamate levels under glutamate and glutamine-free conditions [13]. Exchange of intracellular glutamate for extracellular cystine occurs through the action of the x-C-type transporter (xCT). As the precursor of intracellular cysteine, cystine is necessary to replenish glutathione. Thus, it follows that riluzole treatment could lead to increased oxidative stress, DNA damage, and cell death. Similar mechanisms have not been evaluated for the noncompetitive GRM1 inhibitor BAY 36-7620 where BAY 36-7620-induced receptor inhibition results in reduced glutamate release [14]. Therefore, if the functional mechanism of both drugs is through inhibition of glutamate release and glutamate signaling through GRM1, then functional effects would also be similar.

Both riluzole and BAY 36-7620 negatively regulate the MAPK and Akt signaling pathways in melanoma cell lines, effectively inhibiting cell growth, proliferation, and invasion [14–16]. A phase 0/I trial of riluzole in patients with stage III/IV melanoma demonstrated a correlation between reduced extracellular signal–regulated kinase (ERK) and Akt phosphorylation with reduction in tumor size [17]. Additionally, combined riluzole and ionizing radiation treatment in GRM1-expressing melanoma cell lines and melanoma xenografts in mice yielded synergistic suppression of cell growth and tumor progression as compared to radiation alone [18, 19].

Growing evidence supports the role of glutamate signaling in breast cancer. Consistent with higher GRM1 expression in malignant as compared to normal prostate tissue [20], a significantly higher fraction of human breast tumors express GRM1 as compared to normal breast tissue [1]. Moreover, treatment of estrogen receptor positive (ER+) MCF-7 xenografts with riluzole alone and with an Akt inhibitor suppresses tumor growth *in vivo* [21]. Others have also shown that pharmacologic modulation of glutamate signaling in ER negative, progesterone receptor negative, and human epidermal growth factor receptor 2 (HER2) negative breast cancer cells induces apoptosis, inhibits angiogenesis, and reduces tumor cell growth *in vitro* and *in vivo* [4–6]. These data suggest that riluzole may hold promise as a novel therapeutic agent for the treatment of cancer including all molecular subtypes of breast cancer [1, 4–6, 21].

The cellular and molecular consequences of pharmacologic modulation of glutamate signaling pathways have not yet been fully elucidated in the setting of breast cancer. Nor is the functional target of riluzole fully understood. For example, glutamate plays a critical role in cellular metabolism. Pharmacologic disruption of glutamate levels, e.g. through altered conversion to α-ketoglutarate in the citric acid cycle, can subsequently alter cell bioenergetics, biochemical equilibrium, and metabolic activity affecting cancer cell survival. However, the potential role of riluzole in altering cancer cell metabolism is currently unknown. Moreover, riluzole effects may be tissue-specific due to differing molecular alterations and pathway dysregulation. Therefore, a study was undertaken to investigate the functional actions of riluzole, in comparison to the known noncompetitive GRM1 inhibitor BAY 36-7620, on a molecularly diverse panel of breast cancer cells. This panel of breast cancer cell lines was treated with each glutamate signaling modulator, and the functional effects on cell proliferation, gene expression, cell cycle alterations, DNA damage, and cell metabolism were evaluated.

## RESULTS

### Breast cancer cell lines express GRM1

**Figure 1:**
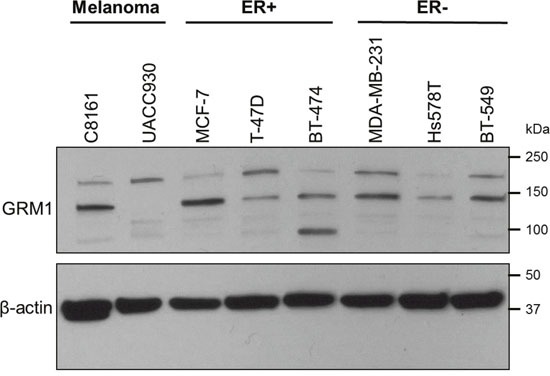
A panel of breast cancer cell lines expresses GRM1 Estrogen receptor (ER) positive (MCF-7, T-47D, BT-474) and ER negative (MDA-MB-231, Hs578T, BT-549) breast cancer cell lines were tested for GRM1 expression by Western blot. C8161 (GRM1+) and UACC930 (GRM1 C-terminal truncation) melanoma cells were included as a positive and negative control, respectively, for GRM1 expression at the predicted molecular weight (MW) of 132 kilodaltons (kDa). β-actin served as a loading control.

ER positive and negative breast cancer cell lines were evaluated for GRM1 expression by Western blot (Figure 1). Each cell line expressed GRM1 but expression was variable across this molecularly distinct set of cell lines: MCF-7, MDA-MB-231, and BT-549 had high expression of GRM1; T-47D, BT-474, and Hs578T had low expression (Table 1).

**Table 1:** Molecular characteristics and drug response of breast cancer cell lines

Cell line	ER status	GRM1 expression	RiluzoleIC_50_-μM (SD)^*^	BAY 36-7620IC_50_-μM (SD)
MCF-7	Positive	High	34.7 (9.9)	27.7 (5.8)
T-47D	Positive	Low	32.6 (4.1)	37.1 (7.6)
BT-474	Positive	Low	24.2 (4.9)	20.8 (6.6)
MDA-MB-231	Negative	High	62.4 (10.9)	41.0 (3.2)
Hs578T	Negative	Low	19.0 (2.7)	21.0 (2.8)
BT-549	Negative	High	24.7 (10.6)	15.7 (1.3)

*Values in parentheses represent +/− standard deviation

### Riluzole and BAY 36-7620 inhibit breast cancer cell growth

**Figure 2:**
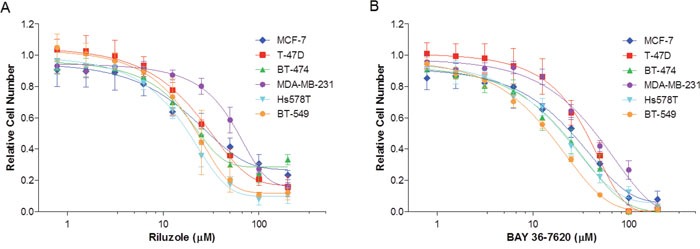
Riluzole and BAY 36-7620 inhibit growth of both hormone receptor positive (MCF-7, T-47D, BT-474) and negative (MDA-MB-231, Hs578T, BT-549) breast cancer cell lines Cells were treated with riluzole **(A)** or BAY 36-7620 **(B)** for 72 h. Relative cell number was measured by MTS assay and normalized relative to vehicle control (DMSO). Data are represented as mean +/− SD.

GRM1 has previously been reported to play a role in breast cancer cell growth and proliferation [1, 4]. To determine the effects of these drugs on cell growth, ER+ and ER- breast cancer cell lines were treated with either riluzole or BAY 36-7620 for 72 h. Both drugs inhibited the number of viable cells in all cell lines (Figure 2A and 2B). IC_50_ values for riluzole and BAY 36-7620 ranged from 19.0-62.4 μM and 15.7-41.0 μM, respectively (Table 1). BT-474, Hs578T, and BT-549 cells were the most sensitive to both drugs while MDA-MB-231 cells were the least sensitive. BAY 36-7620 at the highest concentrations completely inhibited cell growth. At the highest concentrations evaluated, riluzole inhibited cell growth by 70-90% compared to control.

### Treatment with riluzole or BAY 36-7620 inhibits cell proliferation

**Figure 3:**
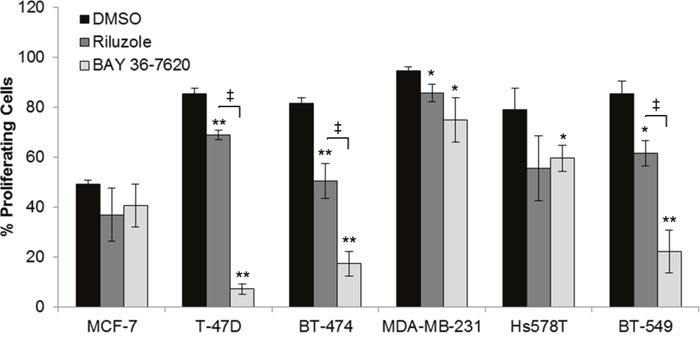
Riluzole and BAY 36-7620 inhibit cell proliferation in both ER+ and ER- breast cancer cell lines Cells were treated with 50 μM riluzole or 50 μM BAY 36-7620 for 72 h. 5-ethynyl-2´-deoxyuridine (EdU) incorporation as a measure of proliferating cells was detected by flow cytometry. Data are represented as mean +/− SD. *P < 0.05 compared to DMSO control. **P < 0.005 compared to DMSO control. ‡ *P* < 0.05 for riluzole compared to BAY 36-7620 treatment (right bracket arm) using one-way ANOVA with Bonferroni's multiple comparison test.

Since both drugs reduced cell number, their effect on proliferation was determined as measured by 5-ethynyl-2´-deoxyuridine (EdU) incorporation. The percentage of proliferating cells was decreased in each breast cancer cell line by riluzole or BAY 36-7620 (Figure 3). However, no association between the anti-proliferative effect of riluzole or BAY 36-7620 and GRM1 levels was observed. T-47D, BT-474, and BT-549 cells were significantly more sensitive to BAY 36-7620 than to riluzole at the concentration evaluated suggesting that BAY 36-7620 has a more potent effect on cell proliferation. Notably, the low GRM1-expressors were still very sensitive to riluzole or BAY 36-7620 implying that there may be off-target effects for each drug.

### Riluzole and BAY 36-7620 alter gene expression signatures in cell cycle and oncogenic pathways

**Figure 4:**
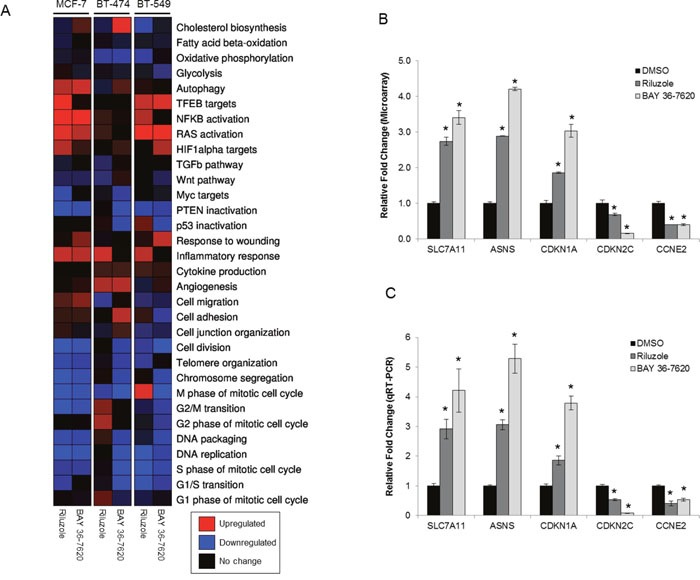
Gene expression signatures are altered by treatment with riluzole or BAY 36-7620 **(A)** RNA from MCF-7 (48 h treatment), BT-474 (24 h treatment), and BT-549 (24 h treatment) cells treated with either 50 μM riluzole or BAY 36-7620 was applied to a gene expression microarray. Three biological replicates were used for each condition. Heat map representation of gene expression signatures from the average of three biological replicates: significantly upregulated (P< 0.05; red), significantly downregulated (P< 0.05; blue), or no significant change (black) compared to expression in cells treated with DMSO control. **(B)** Graphical representation of a selection of genes altered by riluzole or BAY 36-7620 in the gene expression microarray for BT-549 cells compared to DMSO control. **(C)** A two-step RT-PCR was performed on RNA from BT-549 cells for genes up- (SLC7A11, ASNS, CDKN1A) or down-regulated (CDKN2C, CCNE2) by gene expression microarray. Gene expression is shown as relative fold change compared to DMSO control. Data are represented as mean +/− SD. *P < 0.005 compared to DMSO control.

Gene expression analysis of MCF-7, BT-474, and BT-549 cells was done to identify gene sets in pathways altered by either drug to better understand their mechanism of action. These cell lines were included to compare cells with a range of sensitivity to riluzole or BAY 36-7620 with BT-549 being the most sensitive and MCF-7 being the least sensitive. As BT-549 and BT-474 cells are more sensitive to cell death by both drugs, these cell lines were treated for 24 h, whereas the less sensitive MCF-7 cells were treated for 48 h. Overall, riluzole or BAY 36-7620 induced similar gene signature profiles for each of the three cell lines as compared to dimethyl sulfoxide (DMSO) control (Figure 4A). However, differential expression signatures were observed for riluzole as compared to BAY 36-7620. For example, BAY 36-7620, but not riluzole, induced the cholesterol biosynthesis gene signature in MCF-7 and BT-474 cells. Expression signatures for cell cycle genes showed significant decreases in gene expression by both drugs in comparison to DMSO control in MCF-7 and BT-549 cell lines (Figure 4A and 4B, Supplementary Table 1). There are trends of activation of oncogenic pathways such as signatures for hypoxia-inducible factor 1-alpha (HIF1-α), RAS, and nuclear factor kappa-light-chain-enhancer of activated B cells (NFκB) and alteration of tumor suppressor pathways such as signatures for p53 and phosphatase and tensin homolog (PTEN). There is also a trend toward inactivation of the Myc targets signature which is consistent with the observed reduction in cell proliferation (Figure 3). Regarding metabolic signatures, a trend toward up-regulation of TFEB targets is observed in MCF-7 cells treated with riluzole and BT-549 cells treated with either drug, suggesting induction of lysosomal biogenesis and activation of autophagy. Riluzole increased the cell migration gene signature in MCF-7 cells but decreased it in BT-549 cells. It is important to note that differences in gene expression profiles across cell lines may be due to cell line heterogeneity. BT-474 cells are HER2+ and may be under higher replicative stress which may contribute to the variability in a subset of the expression profiles between cell lines.

Gene microarray results were validated using quantitative reverse transcription PCR (qRT-PCR) on a subset of genes found to be up- or down-regulated. A set of cell cycle regulated genes (CDKN1A, CDKN2C, CCNE2) were validated since both riluzole and BAY 36-7620 induced alterations in expression signatures for cell cycle genes and because of the previously observed effects of riluzole on G2/M arrest [21]. Additionally, SLC7A11 gene expression was validated as this gene encodes for the glutamate antiporter xCT. Finally, expression of ASNS, a gene that encodes for the metabolic enzyme asparagine synthetase, was confirmed as glutamate is an important metabolic factor. Comparison between microarray analysis and qRT-PCR showed that the direction of change in gene expression was concordant among all genes tested in BT-549 and BT-474 cells and several genes in MCF-7 cells (Figure 4B and 4C, Supplementary Table 2, Supplementary Figure 1). However, the fold changes that were dissimilar between the two methods may be, in part, due to inherent differences between the methods.

### Riluzole more strongly induces G2/M cell cycle arrest than BAY 36-7620

**Figure 5:**
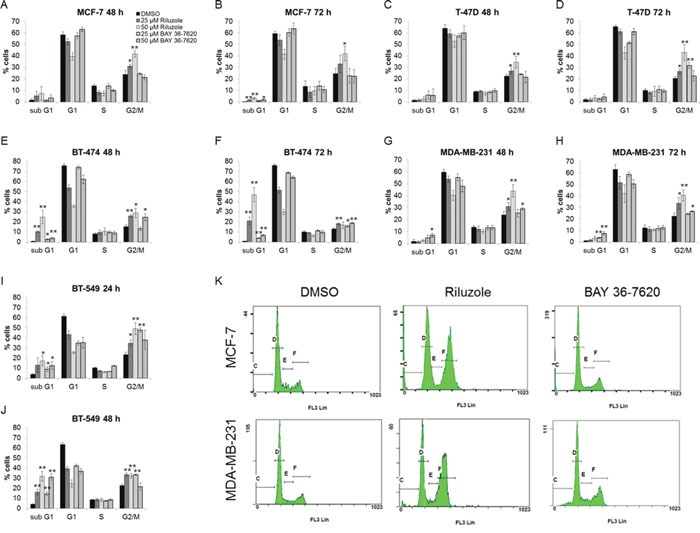
Riluzole induces G2/M cell cycle arrest to a greater extent than BAY 36-7620 MCF-7 **(A, B)**, T-47D **(C, D)**, BT-474 **(E, F)**, MDA-MB-231 **(G, H)**, and BT-549 **(I, J)** cells were treated with either 25 or 50 μM riluzole or BAY 36-7620 for 24, 48, or 72 h. DNA content was measured by flow cytometry. Data are represented as mean +/− SD. *P < 0.05 compared to DMSO control. **P < 0.005 compared to DMSO control. Representative histograms are shown for 50 μM drug treatment after 72 h for MCF-7 and MDA-MB-231 cell lines **(K)**.

Effects of riluzole and BAY 36-7620 on cell cycle distribution were investigated. Each breast cancer cell line treated with riluzole showed a significant dose- and time-dependent induction of G2/M arrest (Figure 5). BT-474 and BT-549 cells were most sensitive to riluzole with an increase in the sub G1 population as early as 48 h and 24 h respectively (Figure 5E and 5I). BAY 36-7620 induced a more modest G2/M arrest in T-47D, BT-474, MDA-MB-231, and BT-549 cell lines (Figure 5D-5J) but had no effect in MCF-7 cells (Figure 5A and 5B) as compared to riluzole. Although both riluzole and BAY 36-7620 inhibited proliferation, more pronounced G2/M arrest by riluzole may implicate other targets beyond those of BAY 36-7620.

### Riluzole but not BAY 36-7620 induces mitotic arrest in breast cancer cells

**Figure 6:**
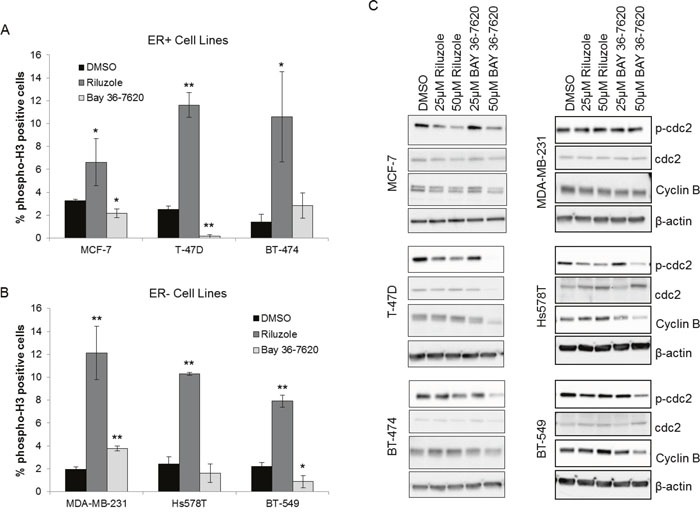
Riluzole induces mitotic arrest in breast cancer cells ER+ **(A)** and ER- **(B)** breast cancer cells were treated with 50 μM riluzole or 50 μM BAY 36-7620 for 48 h. Cells were stained with an antibody specific to phospho-histone H3 at Ser10 (phospho-H3) as a marker for mitosis and detected by flow cytometry. DMSO was used as a vehicle control. *P < 0.05 compared to DMSO control. **P < 0.005 compared to DMSO control. Data are represented as mean +/− SD. **(C)** Phospho-cdc2 (Tyr 15; p-cdc2) and cyclin B1 expression were measured by Western blot after 24 h (Hs578T, BT-549) or 48 h (MCF-7, T-47D, BT-474, MDA-MB-231) drug treatment. Representative blot is shown (Western blot densitometry shown in Supplementary Figure 2). β-actin and cdc-2 served as loading controls for cyclin B1 and p-cdc2 respectively.

To distinguish whether riluzole or BAY 36-7620 treatment induces G2 arrest or mitotic arrest in breast cancer cells, the fraction of cells with phosphorylation of histone H3 was utilized as a marker of mitosis. Riluzole significantly increased the number of phospho-H3 stained cells compared to control in all cell lines suggesting that riluzole induced mitotic arrest (Figure 6A and 6B). In contrast, BAY 36-7620 exhibited variable cell line-dependent effects. BAY 36-7620 significantly decreased the number of phospho-H3 stained cells in MCF-7, T-47D and BT-549 cells while a modest increase was observed in MDA-MB-231 cells (Figure 6A and 6B).

Breast cancer cells treated with riluzole or BAY 36-7620 were also investigated for changes in known markers for mitosis. Riluzole significantly decreased phospho-cdc2 in MCF-7, T-47D, Hs578T, and BT-549 cells and significantly increased cyclin B1 in Hs578T cells with a trend toward increase in T-47D, BT-474, and BT-549 cells (Figure 6C, Supplementary Figure 2). This supports a role for riluzole in induction of mitotic arrest. BAY 36-7620 only decreased phospho-cdc2 in MCF-7, T-47D, Hs578T, and BT-549 cells at higher, more cytotoxic concentrations and did not increase cyclin B levels (Figure 6C, Supplementary Figure 2). The effects of riluzole on phospho-H3, phospho-cdc2, and cyclin B1 suggest that riluzole induces mitotic arrest within G2/M arrest whereas BAY 36-7620 had a minimal effect on both G2/M arrest and more specifically mitotic arrest.

### DNA damage is observed after treatment with BAY 36-7620

**Figure 7:**
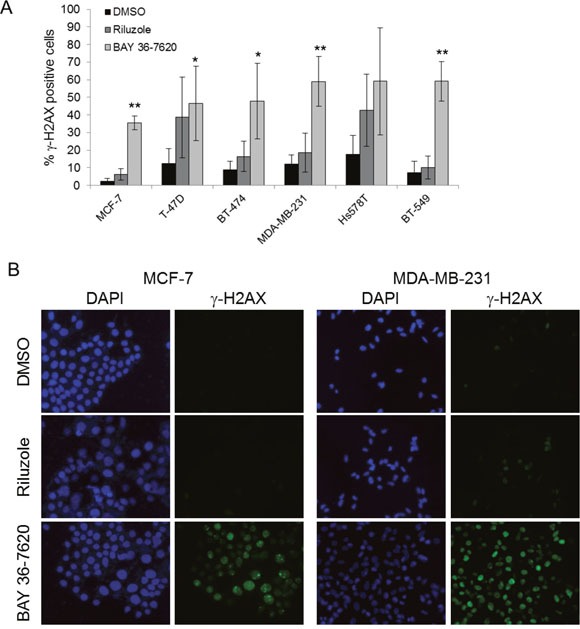
Riluzole and BAY 36-7620 induce DNA damage **(A)** Immunofluorescence for γ-H2AX was performed on cell lines treated with 50 μM riluzole or 50 μM BAY 36-7620 for 24 h. Positive γ-H2AX staining is shown as a percentage of total cells. DAPI was used as a nuclear stain. Data are represented as mean +/− SD. *P < 0.05 compared to DMSO control. **P < 0.005 compared to DMSO control. **(B)** Representative images of MCF-7 and MDA-MB-231 cells with γ-H2AX foci by immunofluorescence.

DNA damage is known to result in G2/M arrest within the cell cycle [22, 23]. To determine whether riluzole or BAY 36-7620 induces DNA damage as a potential cause of G2/M arrest, phosphorylation of histone H2AX (γ-H2AX) was evaluated as a well-described marker of DNA damage, specifically DNA double strand breaks. All breast cancer cell lines treated with either riluzole or BAY 36-7620 had an increased percentage of cells positive for γ-H2AX foci as detected by immunofluorescence (Figure 7A). However, BAY 36-7620 induced a significantly more robust H2AX phosphorylation than riluzole. Increased γ-H2AX nuclear foci after drug treatment can be seen in representative images from an ER+ (MCF-7) and ER- (MDA-MB-231) cell line (Figure 7B).

### Riluzole-induced cell cycle arrest is independent of oxidative stress

**Figure 8:**
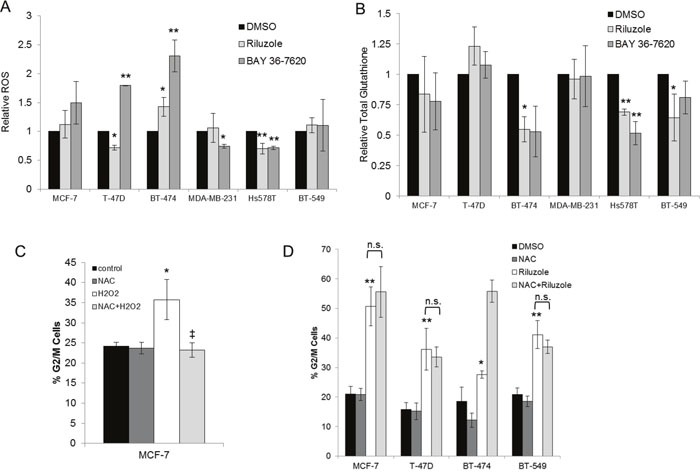
Riluzole-mediated G2/M arrest is independent of induction of oxidative stress Breast cancer cell lines treated with 50 μM riluzole or 50 μM BAY 36-7620 for 24 h were evaluated for levels of reactive oxygen species (ROS) **(A)** and total intracellular glutathione (GSH) **(B)**. Relative fold change was compared to DMSO control. *P < 0.05 compared to DMSO control. **P < 0.005 compared to DMSO control. Cell cycle distribution (represented as the percentage of cells in G2/M) was detected by flow cytometry in cells pretreated with 5mM N-acetyl-cysteine (NAC) followed by the addition of 25 μM H_2_O_2_ **(C)** or 50 μM riluzole **(D)**. *P < 0.05 compared to control. **P < 0.005 compared to control. ‡ *P* < 0.005 for NAC+H_2_O_2_ compared to H_2_O_2_ treatment alone. Not significant (n.s.). Data are represented as mean +/− SD.

It has been hypothesized that riluzole induces oxidative stress due to reduced antiport of glutamate and cystine via xCT, leading to depletion of glutathione stores then DNA damage in melanoma cells [24]. To evaluate if riluzole or BAY 36-7620 increased oxidative stress in breast cancer cells, levels of reactive oxygen species (ROS) and total intracellular glutathione (GSH) were evaluated. BAY 36-7620 significantly increased ROS in T-47D and BT-474 ER+ breast cancer cell lines, while riluzole resulted in significantly increased ROS only in BT-474 cells (Figure 8A). Although the increase in ROS by BAY 36-7620 was not statistically significant in MCF-7 cells, the trend was similar to the other two ER+ cell lines. When comparing the two drugs, the increase in ROS was significantly higher with BAY 36-7620 as compared with riluzole. Neither drug significantly increased ROS in ER- cells. Interestingly, there was a modest *decrease* in ROS in MDA-MB-231 treated with BAY 36-7620 and Hs578T ER- cell lines treated with either riluzole or BAY 36-7620. Total glutathione (GSH) levels decreased after riluzole or BAY 36-7620 treatment in BT-474 and Hs578T cells while no significant effect was observed in the other cell lines (Figure 8B). Both cell lines have relatively low GRM1 protein levels suggesting that drug treatment may affect other glutamate receptor targets.

To determine whether riluzole-mediated cell cycle arrest is dependent on induction of oxidative stress, cells were pretreated with the ROS scavenger N-acetyl-cysteine (NAC). As a positive control for ROS, MCF-7 cells were treated with hydrogen peroxide (H_2_O_2_). This led to induction of G2/M cell cycle arrest which was prevented by pretreatment with NAC (Figure 8C). However, NAC pretreatment did not prevent riluzole-induced G2/M cell cycle arrest in MCF-7, T-47D, BT-474, and BT-549 cells suggesting that riluzole exerts its effect independent of ROS generation (Figure 8D).

### Riluzole alters cellular metabolism

**Figure 9:**
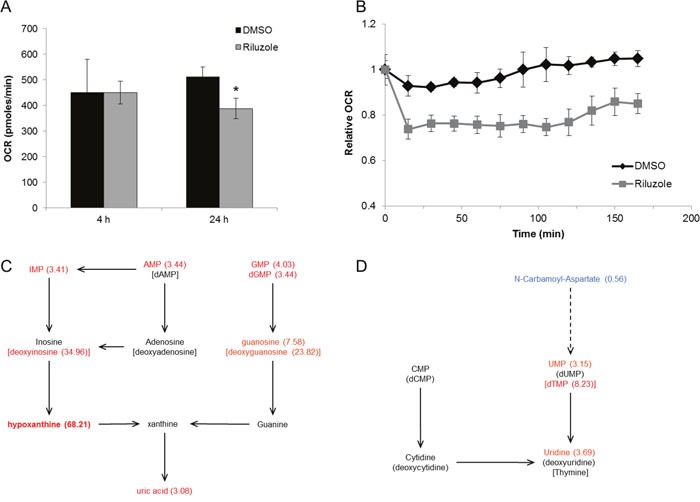
Riluzole inhibits oxidative phosphorylation and alters cellular metabolism **(A)** BT-474 cells were treated with control (DMSO) or 50 μM riluzole for 4 h or 24 h. Oxygen consumption rates (OCR) were measured on the XF analyzer and are shown relative to basal levels. *P < 0.05 compared to DMSO control. **(B)** Basal OCR was measured in BT-474 cells at time 0 followed by treatment with DMSO or 50 μM riluzole. OCR was measured at 15 min intervals after treatment. Data are represented as mean +/− SD. **(C, D)** Cellular metabolite analysis by LC/MS was performed on BT-474 cells treated with 50 μM riluzole for 24 h and normalized to DMSO control (see Supplementary Table 3 for complete list of relative fold changes). Components of purine **(C)** and pyrimidine **(D)** metabolism altered by riluzole are shown (fold change in parenthesis).

Riluzole has been shown to alter intracellular glutamate levels. As glutamate is a critical component of the citric acid cycle, metabolic alterations were evaluated as a possible contributor to riluzole-induced cell cycle arrest. BT-474 cells were used because this cell line is particularly sensitive to riluzole with respect to cell cycle arrest compared to other cell lines tested. Oxygen consumption rate (OCR) was evaluated at short and long time intervals as a measure of oxidative phosphorylation in riluzole-treated cells. OCR was unchanged after 4 h treatment, but levels of oxidative phosphorylation significantly decreased after 24 h riluzole treatment compared to control suggesting that riluzole inhibits oxidative metabolism in this breast cancer cell line (Figure 9A). To investigate the immediate effect of riluzole on oxidative phosphorylation, BT-474 cells were treated with riluzole followed by real-time detection of OCR at short intervals. Riluzole induced an immediate reduction in OCR and inhibition of return to basal OCR levels over the time frame evaluated as compared to control (Figure 9B).

To identify which key metabolic pathways were altered, water-soluble metabolites from riluzole-treated BT-474 cells were measured by liquid chromatography–mass spectrometry (LC/MS). Cells treated with riluzole have a significantly altered metabolic profile, i.e. riluzole induced a greater than two-fold increase in the cellular levels of several nucleotides and nucleosides (Supplementary Table 3). Intermediary nucleoside levels are the most significantly increased upon riluzole treatment suggesting altered purine and pyrimidine catabolism (Figure 9C and 9D). Furthermore, N-carbamoyl-aspartate, an intermediate in *de novo* pyrimidine synthesis, was the only metabolite with significantly reduced levels upon riluzole treatment suggesting riluzole may inhibit *de novo* pyrimidine synthesis.

## DISCUSSION

Incorporation of riluzole into breast cancer treatment paradigms has been hampered by a limited understanding of its mechanism of action. In this study, a pharmacologic approach was undertaken to investigate the antitumor effects of riluzole in a panel of human breast cancer cell lines and compared to the effects of the known glutamate receptor antagonist BAY 36-7620. Treatment with either drug produced cell line-dependent effects on markers for proliferation, cell cycle, and DNA damage. Both drugs inhibited cell growth and cell number while altering expression of genes involved in cell cycle regulation and oncogenic pathways. While riluzole and BAY 36-7620 both induce cell death, they have differential effects within cell cycle. Whereas riluzole induced cell death with mitotic arrest, BAY 36-7620 caused cell death without substantial effect on cell cycle. Riluzole induced significant metabolic changes in the cell including decreased oxidative phosphorylation and alteration of cellular metabolite levels suggesting a novel role for riluzole in cell metabolism. Some cell lines showed differential sensitivity to either riluzole or BAY 36-7620. These data support distinct drug-induced mechanisms of cell cycle inhibition leading to cell death.

Riluzole-induced DNA damage in breast cancer cell lines was variable and cell line-specific, an observation consistent with that in melanoma cells [24]. However, BAY 36-7620 had a significantly more robust effect as compared to riluzole. Genomic variability between breast cancer cell lines may influence susceptibility to riluzole. One such area of speculation includes TP53 mutational status or HER2 status that place cells under replicative stress. Riluzole did not induce DNA damage in MCF-7 cells (TP53 wildtype) whereas moderate induction of DNA damage occurred in the majority of TP53 mutant cell lines. In contrast, BAY 36-7620 had a more universal DNA damaging effect on all cell lines tested, again suggesting a distinct mechanism from riluzole. Interestingly, riluzole-induced mitotic arrest was more prominent in TP53-mutated cell lines as compared to TP53 wildtype MCF-7 cells suggesting that loss of p53 checkpoint activity may lead to mitotic catastrophe in TP53 mutant cells. Given the paucity of TP53 wildtype breast cancer cell lines, this relationship is difficult to confirm. Further exploration of the role of p53 may enlighten any potential role in response to riluzole. In addition to TP53 mutations, alterations in other genes involved in the DNA damage response or other pathways may also sensitize cells to riluzole-induced DNA damage. For example, BT-474 cells have alterations in BRCA2, ATM, and PARP1 that may predispose these cells to increased DNA damage by riluzole. Also, BT-474 cells are Her2 overexpressing and have a higher mitotic rate which may induce replicative stress upon treatment with riluzole. Further exploration of drug response in genetically engineered cell lines may help to better delineate these relationships.

While others have found correlations between riluzole-induced DNA damage and GRM1 expression in melanoma cell lines [24], there was no apparent correlation between GRM1 level and drug response to riluzole in these breast cancer cell lines. Moreover, riluzole was still effective in cell lines with the lowest GRM1 expression suggesting that riluzole may affect non-GRM1 targets. Similarly, Speyer et al. [25] found that genetically altering GRM1 expression in breast cancer cells did not affect response to riluzole. These data and that by others are consistent with the hypothesis that riluzole may have effects that are independent of direct signaling through GRM1. Neither data eliminate a possible role for other glutamate receptors as mediators of riluzole response in these cell lines and would require further study.

Others have shown that riluzole alters redox homeostasis as a mechanism for DNA damage and cell death in melanoma cells *in vitro* [24]. Increased ROS and reduced GSH were observed in a subset of riluzole-treated breast cancer cell lines suggesting alteration of the redox state of some cell lines. However, the antioxidant NAC was unable to inhibit riluzole-induced cell cycle arrest suggesting that either these two events are not coupled or that DNA damage occurs as a later event. While riluzole induced oxidative stress, this seemed to be independent of its effect on cell cycle in breast cancer cells.

Riluzole has been shown to indirectly inhibit GRM1 activity in melanoma [13]. The data presented here suggest that there are distinct drug-specific effects between riluzole and BAY 36-7620 in breast cancer cells. Further, these data are consistent with effects that may be independent of direct signaling through GRM1. These effects include altered oxidative metabolism and levels of cellular metabolites. Riluzole effect on other glutamate receptors or pathways regulated by glutamate cannot be ruled out. However, ammonia levels, which can be produced by glutamate metabolism, were unchanged by riluzole (data not shown) suggesting that it does not induce ammonia toxicity as its mechanism of action. As other pathways are glutamate-dependent, including but not limited to amino acid biosynthesis and lipid synthesis, additional research into the effects of riluzole on these pathways is warranted.

Riluzole also altered precursors or derivatives of nucleotides that compose DNA. As such, altered DNA biosynthesis pathways remain a possible target. It has been shown that incorporation of precursor nucleotides in DNA leads to stalled replication forks and ultimately cell cycle arrest and death [26]. Alternatively, an increase in nucleotide catabolism may not be a direct effect of riluzole treatment, but a consequence of mitotic arrest. Riluzole-induced mitotic arrest shifts the cell population towards an increase in the fraction of cells in G2/M. Cells in G2/M may increase the catabolism of nucleotides that are not further needed for DNA synthesis. The observed increase in nucleotide/nucleoside levels may reflect cell cycle-specific differences.

Riluzole has been studied in Phase II clinical trials for the treatment of patients with stage III and IV melanoma with 42% of patients exhibiting stable disease [27]. As an FDA-approved drug for other indications, riluzole could be quickly moved into trials for the treatment of breast cancer based on its antitumor effects in preclinical models. Understanding the mechanistic effects provides a rationale to explore the use of riluzole alone or in combination with other anti-cancer therapies. Breast cancer subtypes with high replicative stress (e.g. TP53 mutation, MYC amplification, high mitotic index) or those with DNA repair defects (e.g. triple negative breast cancers, BRCA-related breast cancers) may represent markers for more suitable patient selection for riluzole-based regimens. However, this would require additional biomarker evaluation to determine association with riluzole sensitivity. Based on this study and others, further development of riluzole and direct glutamate signaling modulators for preclinical and clinical studies is warranted.

## MATERIALS AND METHODS

### Cell lines and culture

MCF-7, T-47D, BT-474, MDA-MB-231, Hs578T, and BT-549 breast cancer cell lines (Charles River Laboratories, Inc, New York, NY; ATCC, Manassas, VA) were maintained in Dulbecco's Modified Eagle's medium (DMEM; Sigma Aldrich, St. Louis, MO) plus 10% fetal bovine serum (FBS) and 1% penicillin-streptomycin (Life Technologies, Grand Island, NY) at 37°C in a humidified atmosphere containing 5% CO_2_. All cell lines were authenticated by the Gene Expression and Genotyping Core Facility at the University of Florida Interdisciplinary Center for Biotechnology Research using the established STR profiling system.

### Reagents and antibodies

Riluzole hydrochloride (Tocris Bioscience, Bristol, UK) and BAY 36-7620 (Tocris Bioscience, Bristol, UK) were dissolved in DMSO. GRM1 primary antibody is specific to the C-terminal region of human GRM1α and was used at a dilution of 1:2000 for Western blotting (catalog number 36350002; Novus Biologicals, Littleton, CO). Additional primary antibodies for Western blotting or immunofluorescence include monoclonal anti-ß-actin clone AC-15 (Sigma Aldrich, St. Louis, MO); phospho-cdc2 (Tyr15), cyclin B1, phospho-histone H2AX (Ser139) (Cell Signaling, Danvers, MA). Primary antibodies for flow cytometry include phospho-histone H3 (Ser10) rabbit monoclonal (Alexa Fluor^®^ 488 conjugate), histone H3 rabbit monoclonal (Alexa Fluor^®^ 647 conjugate), rabbit monoclonal IgG isotype control (Alexa Fluor^®^ 488 conjugate), rabbit monoclonal IgG isotype control (Alexa Fluor^®^ 647 conjugate) (Cell Signaling, Danvers, MA).

### Western blot analysis

Cell lysates were prepared using RIPA buffer (Sigma Aldrich, St. Louis, MO) with Protease Inhibitor Cocktail and Phosphatase Inhibitor Cocktail 2 and 3 (Sigma Aldrich, St. Louis, MO) and centrifugation at 12,000 X *g* for 15 min at 4°C. Protein concentration was determined using the Bradford protein assay reagent (Bio-Rad, Hercules, CA). Lysates were separated by 4-20% gradient sodium dodecyl sulphate-polyacrylamide gel electrophoresis (SDS-PAGE) under reducing conditions. Protein was transferred onto a polyvinyl difluoride (PVDF) membrane. Membranes were incubated overnight at 4°C with primary antibody followed by incubation with horseradish peroxidase secondary antibody. Enhanced chemiluminescence (ECL) reagent was added to the membrane according to the manufacturer's protocol (Pierce Biotechnology Inc., Rockford, IL). Chemiluminescence was detected on the ChemiDoc™ Imaging System, and densitometry was performed using the Image Lab software (Bio-rad, Hercules, CA)

### Drug sensitivity assay

Cells were seeded in 96 well plates and allowed to attach overnight. Cells were then treated with increasing concentrations of drug. After 72h of drug treatment, MTS reagent (Promega, Madison, WI) was added and cells were incubated at 37°C for 2-4 h. Absorbance was measured at 490 nm using a Wallac 1420 Victor^3^ plate reader (Perkin Elmer, Waltham, MA). Viability was expressed as a percentage of control by dividing the absorbance of each treated sample by the average of the untreated controls.

### Cell proliferation assay

Cells were seeded in 6 well plates and allowed to attach overnight. Cells were then treated with drug for 72 h. EdU was added at a final concentration of 10 μM at 48 h drug treatment. Cell number was determined using the Vi-CELL Cell Viability Analyzer (Beckman Coulter, Indianapolis, IN). Equal cell numbers were fixed, permeabilized, and stained according to manufacturer's instructions (Click-iT^®^ EdU Alexa Fluor^®^ 488 Flow Cytometry Assay Kit; Life Technologies, Grand Island, NY). Percentage of proliferating cells was assessed by flow cytometry at an excitation wavelength of 488 nm. Cells without EdU labeling were used as a negative control for proper gating conditions. Data were analyzed using CXP software (Cytomics FC 500 Series; Beckman Coulter, Indianapolis, IN).

### Gene expression analysis

MCF-7 cells were treated with 50 μM riluzole or BAY 36-7620 for 48h. BT-474 and BT-549 cells were treated for 24h due to rapid entry of cells into subG1. Total RNA was purified with RNeasy mini kit following manufacturer's protocol (Qiagen, Germantown, MD). RNA was subjected to DNase treatment to remove contaminating DNA. The Human Genome U133A 2.0 Array was used to measure gene expression changes from drug treatment compared to DMSO control. Three independent replicates were used for each condition. For analysis, raw CEL files were processed using the justRMA function in R Bioconductor, obtaining log2 expression values. Gene expression signatures were analyzed using Gene Set Enrichment Analysis [28], obtaining a quantification of the statistical significance for upregulation (P+) or downregulation (P-) for each signature and sample pair. A sample was said to have a signature significantly upregulated if P+ < 0.05 (red), significantly downregulated if P- < 0.05 (blue), and no significant change otherwise (black). For microarray validation, RNA from cells treated with DMSO, 50 μM riluzole, or 50 μM BAY 36-7620 was reverse transcribed using the Taqman Reverse Transcription kit following manufacturer's protocol (Thermo Fisher Scientific, Waltham, MA). Pre-designed Taqman assays for genes validated were used to perform quantitative PCR on the complementary DNA. The RPLP0 gene was used as a housekeeping gene control. Results are shown as relative fold change of gene expression compared to DMSO control treatment using the delta delta Ct method.

### Cell cycle analysis

Cells grown overnight were then treated with drug for 48 and 72 h. All cells were collected by trypsinization and cell number was determined using the Vi-CELL Cell Viability Analyzer (Beckman Coulter, Indianapolis, IN). Single cell suspensions with equal cell number were prepared. Cells were fixed with absolute ethanol and incubated overnight at -20°C then stained with 10 μg/ml propidium iodide and 100 μg/ml RNase A in phosphate-buffered saline (PBS; Sigma Aldrich, St. Louis, MO). Cell cycle distribution was assessed by flow cytometry and analyzed using CXP software (Cytomics FC 500 Series; Beckman Coulter, Indianapolis, IN).

### Histone H3 phosphorylation

Cells were grown overnight and then treated with drug for 48 and 72 h. Cell number was determined using the Vi-CELL Cell Viability Analyzer (Beckman Coulter, Indianapolis, IN). Paraformaldehyde was added to suspended cells to a final concentration of 4% for 10 min at 37°C. Cells were permeabilized with 90% methanol, incubated on ice for 30 min, and then washed twice with incubation buffer (5 mg/ml of BSA in PBS). Cells were co-stained for 1 h with phospho-histone H3 (Alexa Fluor^®^ 488) and histone H3 (Alexa Fluor^®^ 647) antibodies, evaluated by flow cytometry, and analyzed using CXP software. Unstained and single stained controls were used to gate the cells.

### γ-H2AX immunofluorescence

Cells were grown in chamber slides and then treated with drug for 24 h. Paraformaldehyde was added to cells to a final concentration of 4%. Cells were permeabilized with 0.5% Triton X-100 for 10 min. Primary antibody to γ-H2AX was added to cells in 5% goat serum in PBS (blocking buffer) for 1 h. Cells were washed with PBS and incubated with fluorescein (FITC)-conjugated secondary antibody in blocking buffer for 1 h. Cells were mounted with ProLong Diamond Antifade Mountant with DAPI (Life Technologies, Grand Island, NY) nuclear stain and visualized using the Nikon Eclipse Ti fluorescent microscope. Cells positive for γ-H2AX foci were counted from four independent fields.

### Reactive oxygen species (ROS) detection

Single cell suspensions of 24 h drug-treated cells were incubated with CELLROX green reagent (Life Technologies, Grand Island, NY) for 45 min at 37°C. Oxidation-induced fluorescence was detected by flow cytometry as a measure of ROS and corrected for background autofluorescence. For ROS protection experiments, cells were pretreated with 5 mM N-acetyl-cysteine (NAC; Sigma Aldrich, St. Louis, MO) for 1 h followed by treatment with drug.

### Intracellular glutathione (GSH) measurement

Cells were treated with drug for 24 h and then harvested. Cell pellets were then lysed with 10mM hydrochloric acid followed by two freeze thaws. Total intracellular glutathione was measured following manufacturer's instructions (GSSG/GSH Quantification Kit, Dojindo Molecular Technologies, Rockville, MD).

### Oxygen consumption rate measurement

Oxygen consumption rate (OCR) was measured using the Extracellular Flux Analyzer (XF24, Seahorse Bioscience). For long-term treatments, BT-474 cells were grown in normal growth media overnight in XF24 plates. Cells were then treated with DMSO control or 50 μM riluzole for 4 or 24 h. Media was replaced with DMEM without sodium bicarbonate or FBS and incubated for 1 h. Basal OCR measurements were taken in DMEM without sodium bicarbonate or FBS. For short-term treatments (OCR measurements immediately after injection of DMSO or 50 μM riluzole), cells were grown in normal growth media overnight in XF24 plates then replaced with DMEM without sodium bicarbonate or FBS and incubated for 1 h. OCR measurements were taken at time 0, then every 15 min after injection of DMSO or 50 μM riluzole.

### Cellular metabolite analysis by LC/MS

BT-474 cells were treated with DMSO or 50 μM riluzole for 24 h. Water-soluble metabolites were extracted and analyzed by LC/MS as previously described [29]. Metabolite measurements were normalized to cell number.

### Statistical analysis

All graphical data are represented as mean +/− standard deviation (SD). For riluzole or BAY 36-7620 treatments, unpaired, two-tailed Student's t-test was used to calculate the *P* value of the difference between control (DMSO) and treated cells from three independent experiments where a *P* value of less than 0.05 was considered statistically significant. For comparison of riluzole and BAY 36-7620, one way analysis of variance (ANOVA) with Bonferroni's multiple comparison test was used to calculate statistical significance between groups. For comparison of cellular metabolite levels between control and riluzole, t-test with Bonferroni correction was used where a *P* value of less than 0.05 was considered statistically significant.

## SUPPLEMENTARY MATERIALS FIGURES AND TABLES





## Abbreviations

ANOVA: analysis of variance
ASNS: asparagine synthetase (glutamine-hydrolyzing)
CCNE2: cyclin E2
CDKN1A: cyclin dependent kinase inhibitor 1A
CDKN2C: cyclin dependent kinase inhibitor 2C
CNS: central nervous system
DMEM: Dulbecco's Modified Eagle's medium
DMSO: dimethyl sulfoxide
EdU: 5-ethynyl-2´-deoxyuridine
ER: estrogen receptor
ERK: extracellular signal–regulated kinase
GRM1: metabotropic glutamate receptor 1
GSH: intracellular glutathione
H_2_O_2_: hydrogen peroxide
γ-H2AX: phosphorylation of histone H2AX
HER2: human epidermal growth factor receptor 2
HIF1-α: hypoxia-inducible factor 1-alpha
LC/MS: liquid chromatography-mass spectrometry
MAPK: mitogen-activated protein kinase
MW: molecular weight
NAC: N-acetyl-cysteine
NFκB: nuclear factor kappa-light-chain-enhancer of activated B cells
OCR: oxygen consumption rate
PBS: phosphate-buffered saline
PKC: protein kinase C
PLC: phospholipase C
PTEN: phosphatase and tensin homolog
PVDF: polyvinyl difluoride
qRT-PCR: quantitative reverse transcription PCR
ROS: reactive oxygen species
SD: standard deviation
SDS-PAGE: sodium dodecyl sulphate-polyacrylamide gel electrophoresis
SLC7A11: solute carrier family 7 member 11
TFEB: transcription factor EB
TGF beta: transforming growth factor beta
xCT: x-C-type transporter
