# Supplementary material for: Riluzole exerts distinct antitumor effects from a metabotropic glutamate receptor 1-specific inhibitor on breast cancer cells
Source: Oncotarget. 2017 May 18;8(27):44639–53. doi: 10.18632/oncotarget.17961 (PMC5546507; doi:10.18632/oncotarget.17961)
Supplement: Supplementary file 1 [file oncotarget-08-44639-s001.pdf]

Riluzole exerts distinct antitumor effects from a metabotropic glutamate receptor 1-specific inhibitor on breast cancer cells

SUPPLEMENTARY MATERIALS

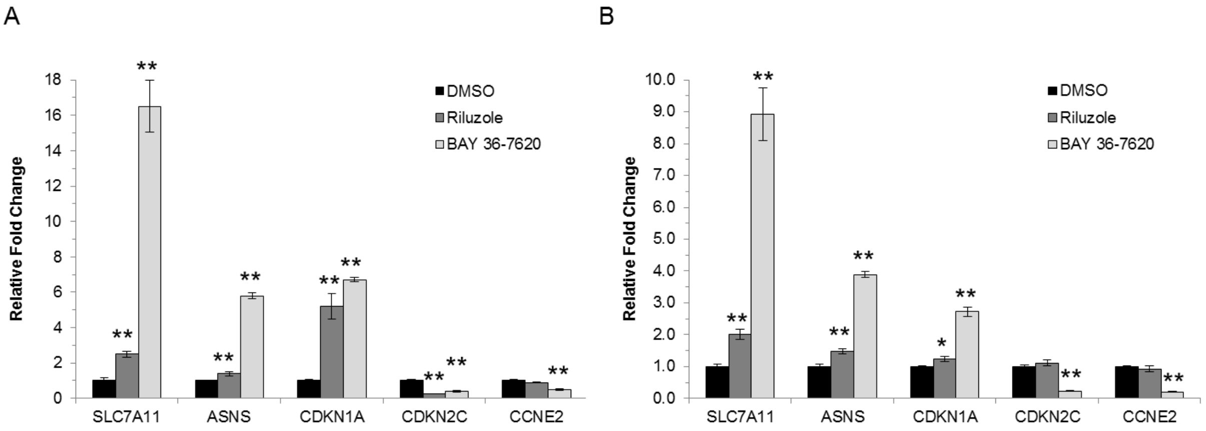

Supplementary Figure 1: RT-PCR validation of gene expression microarray. A two-step RT-PCR was performed on RNA isolated from MCF-7 **(A)** and BT-474 **(B)** cells for genes up- (SLC7A11, ASNS, CDKN1A) or down-regulated (CDKN2C, CCNE2) by gene expression microarray. Gene expression is shown as relative fold change compared to DMSO control. *P < 0.05 compared to DMSO control. **P < 0.005 compared to DMSO control. Data are represented as mean +/- SD.

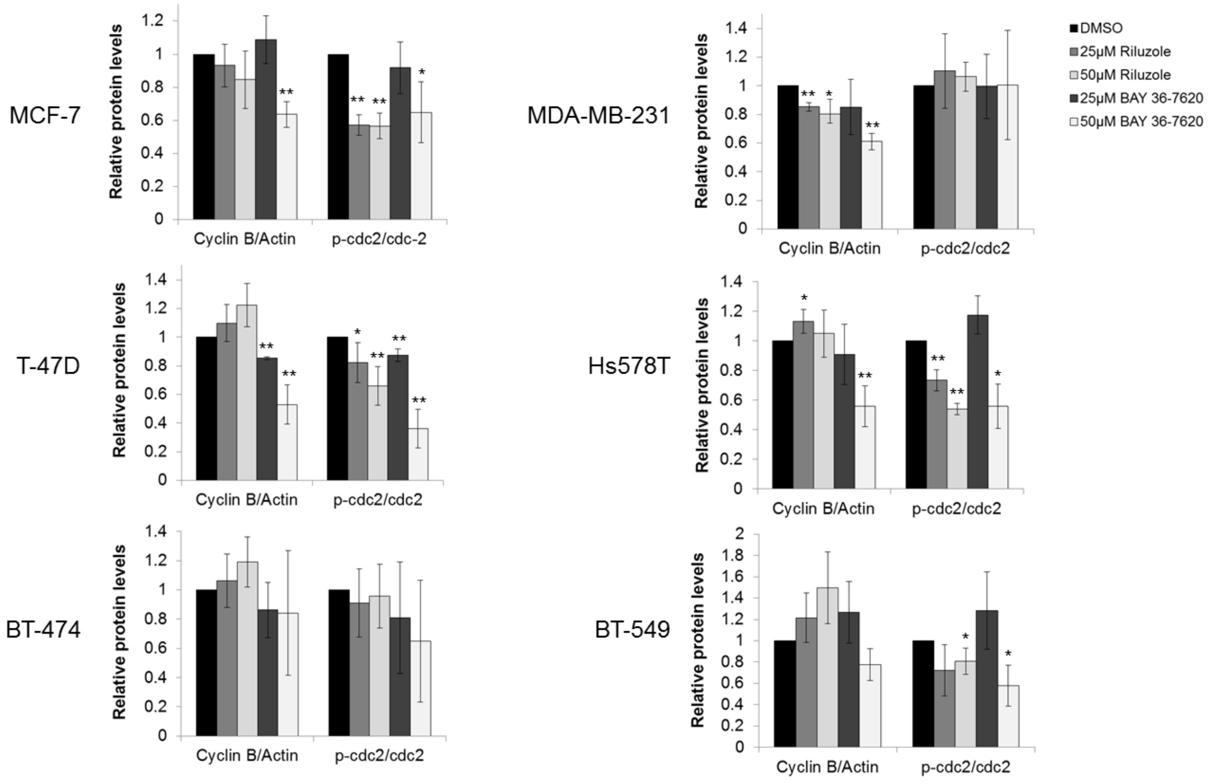

Supplementary Figure 2: Phospho-cdc2 and cyclin B1 expression are significantly altered by treatment with riluzole or BAY 36-7620. Phospho-cdc2 (Tyr 15; p-cdc2) and cyclin B1 expression were measured by Western blot after 24 h (Hs578T, BT-549) or 48 h (MCF-7, T-47D, BT-474, MDA-MB-231) drug treatment. β-actin and cdc-2 served as loading controls for cyclin B1 and p-cdc2 respectively. Densitometry of bands from three independent experiments and normalization to controls are shown. **P* < 0.05 compared to DMSO control. ***P* < 0.005 compared to DMSO control. Data are represented as mean +/- SD.

Supplementary Table 1: Statistical analysis of microarray gene signatures up- or down-regulated by riluzole or BAY 36-7620 (p-values shown)

Gene Signature*	MCF-7		BT-474		BT-549	
	Riluzole	BAY 36-7620	Riluzole	BAY 36-7620	Riluzole	BAY 36-7620
Cholesterol biosynthesis	0.311	0.379	0.344	0.001	0.008	0.205
Fatty acid beta-oxidation	0.318	0.067	0.133	0.274	0.191	0.248
Oxidative phosphorylation	0.234	0.147	0.040	0.025	0.028	0.109
Glycolysis	0.159	0.220	0.150	0.224	0.186	0.556
Autophagy	0.025	0.009	0.286	0.375	0.181	0.110
TFEB targets	< 0.001	0.141	0.067	0.082	0.012	0.003
NFκB activation	< 0.001	< 0.001	0.257	0.135	0.029	0.092
RAS activation	0.002	0.021	0.284	0.082	< 0.001	< 0.001
HIF1 alpha targets	0.025	0.251	0.080	0.268	0.055	0.036
TGF beta pathway	0.254	0.102	0.435	0.123	0.054	0.075
Wnt pathway	0.389	0.392	0.504	0.211	0.068	0.390
Myc targets	0.006	0.084	0.162	0.033	0.056	0.181
PTEN inactivation	< 0.001	0.004	0.099	0.003	0.010	0.024
p53 inactivation	0.054	0.063	0.165	0.007	0.456	0.009
Response to wounding	0.132	0.535	0.127	0.081	0.185	0.015
Inflammatory response	0.013	0.022	0.008	0.098	0.019	0.050
Cytokine production	0.096	0.080	0.302	0.320	0.313	0.235
Angiogenesis	0.108	0.194	0.035	0.024	0.287	0.212
Cell migration	0.373	0.543	0.049	0.124	0.474	0.352
Cell adhesion	0.242	0.268	0.077	0.022	0.239	0.679
Cell junction organization	0.233	0.158	0.246	0.329	0.502	0.364
Cell division	< 0.001	0.002	0.091	0.016	0.044	< 0.001
Telomere organization	0.007	0.023	0.140	0.027	0.044	0.090
Chromosome segregation	< 0.001	0.003	0.142	0.010	0.126	0.009
M phase of mitotic cell cycle	< 0.001	0.001	0.031	< 0.001	0.003	< 0.001
G2/M transition	< 0.001	0.003	0.538	0.080	0.342	0.031
G2 phase of mitotic cell cycle	0.056	0.097	0.046	0.075	0.182	0.049
DNA packaging	0.006	0.010	0.247	0.018	0.264	0.009
DNA replication	< 0.001	< 0.001	0.063	0.001	< 0.001	0.021
S phase of mitotic cell cycle	0.041	0.040	0.114	< 0.001	< 0.001	0.011
G1/S transition	0.030	0.072	0.155	0.002	< 0.001	0.039
G1 phase of mitotic cell cycle	0.095	0.131	0.433	0.349	0.313	0.143

* TFEB:transcription factor EB; NFκB: nuclear factor kappa-light-chain-enhancer of activated B cells; HIF1: alphahypoxia-inducible factor 1-alpha; TGF: beta-transforming growth factor beta; PTEN: phosphatase and tensin homolog

Supplementary Table 2: Validation of microarray gene expression profiling using quantitative RT-PCR

Gene†	MCF-7				BT-474				BT-549			
	Riluzole/DMSO Fold change		BAY 36-7620/DMSO Fold change		Riluzole/DMSO Fold change		BAY 36-7620/DMSO Fold change		Riluzole/DMSO Fold change		BAY 36-7620/DMSO Fold change	
	Microarray	qRT-PCR	Microarray	qRT-PCR	Microarray	qRT-PCR	Microarray	qRT-PCR	Microarray	qRT-PCR	Microarray	qRT-PCR
SLC7A11	1.1 (0.15)*	2.5 (0.18)	1.4 (0.16)	16.5 (1.47)	1.3 (0.17)	2.0 (0.17)	10.0 (1.38)	8.9 (0.83)	2.7 (0.11)	2.9 (0.33)	3.4 (0.19)	4.2 (0.73)
ASNS	5.4 (0.48)	1.4 (0.12)	11.5 (1.08)	5.8 (0.17)	1.2 (0.08)	1.5 (0.07)	4.3 (0.04)	3.9 (0.10)	2.9 (0.01)	3.1 (0.17)	4.2 (0.05)	5.3 (0.49)
CDKN1A	1.0 (0.09)	5.2 (0.70)	1.0 (0.11)	6.7 (0.12)	1.1 (0.09)	1.2 (0.08)	2.7 (0.09)	2.7 (0.15)	1.9 (0.02)	1.9 (0.15)	3.0 (0.18)	3.8 (0.23)
CDKN2C	0.5 (0.01)	0.3 (0.01)	0.2 (0.01)	0.4 (0.04)	1.0 (0.04)	1.1 (0.09)	0.3 (0.03)	0.2 (0.02)	0.7 (0.03)	0.5 (0.03)	0.2 (0.01)	0.1 (0.01)
CCNE2	1.0 (0.05)	0.9 (0.04)	1.1 (0.09)	0.5 (0.04)	0.8 (0.04)	0.9 (0.10)	0.3 (0.02)	0.2 (0.02)	0.4 (0.004)	0.4 (0.08)	0.4 (0.03)	0.5 (0.05)

† SLC7A11: solute carrier family 7 member 11; ASNS: asparagine synthetase (glutamine-hydrolyzing); CDKN1A: cyclin E2

* Values in parentheses represent +/- standard deviation

Supplementary Table 3: Effect of riluzole on water-soluble metabolite levels

See Supplementary File 1
