# Supplementary material for: Riluzole exerts distinct antitumor effects from a metabotropic glutamate receptor 1-specific inhibitor on breast cancer cells
Source: Oncotarget. 2017 May 18;8(27):44639–53. doi: 10.18632/oncotarget.17961 (PMC5546507; doi:10.18632/oncotarget.17961)
Supplement: Supplementary file 2 [file oncotarget-08-44639-s002.docx]

Supplementary Table 3. Effect of riluzole on water-soluble metabolite levels

| **Water-soluble metabolite** | **Riluzole/DMSO fold change** | ***P*-value (upregulated, Bonferroni correction)** | ***P*-value (downregulated, Bonferroni correction)** |
| --- | --- | --- | --- |
| hypoxanthine | 68.21 | < 0.001 | 1.0 |
| deoxyinosine | 34.96 | 0.013 | 1.0 |
| deoxyguanosine | 23.82 | 0.007 | 1.0 |
| dTMP | 8.83 | 0.008 | 1.0 |
| guanosine | 7.58 | 0.008 | 1.0 |
| GMP | 4.03 | 0.003 | 1.0 |
| uridine | 3.69 | 0.010 | 1.0 |
| dTDP | 3.59 | 0.052 | 1.0 |
| AMP | 3.44 | 0.009 | 1.0 |
| dGMP | 3.44 | 0.010 | 1.0 |
| IMP | 3.41 | 0.008 | 1.0 |
| CDP | 3.35 | 0.123 | 1.0 |
| UMP | 3.15 | 0.001 | 1.0 |
| uric acid | 3.08 | 0.029 | 1.0 |
| CDP-ethanolamine | 2.98 | 0.016 | 1.0 |
| 3-hydroxybutyryl-CoA | 2.97 | 0.099 | 1.0 |
| malonyl-CoA | 2.97 | 0.105 | 1.0 |
| CDP-choline | 2.90 | 0.184 | 1.0 |
| dCTP | 2.88 | 0.191 | 1.0 |
| CTP | 2.54 | 0.364 | 1.0 |
| dTTP | 2.34 | 0.340 | 1.0 |
| acadesine | 2.32 | 0.084 | 1.0 |
| cysteine | 2.30 | 1.0 | 1.0 |
| GDP | 2.29 | 0.141 | 1.0 |
| acetyl-CoA | 2.25 | 0.044 | 1.0 |
| IDP | 2.21 | 0.316 | 1.0 |
| adenosine 5'-phosphosulfate | 2.20 | 0.102 | 1.0 |
| ADP | 2.20 | 0.106 | 1.0 |
| N-acetyl-glutamate | 2.13 | 0.123 | 1.0 |
| propionyl-CoA | 2.00 | 0.089 | 1.0 |
| D-glucosamine-6-phosphate | 1.97 | 0.411 | 1.0 |
| acetylphosphate | 1.96 | 1.0 | 1.0 |
| UDP | 1.92 | 0.329 | 1.0 |
| 3-hydroxy-3-methylglutaryl-CoA | 1.83 | 0.107 | 1.0 |
| asparagine | 1.78 | 0.095 | 1.0 |
| NADPH | 1.77 | 0.610 | 1.0 |
| butyryl-CoA | 1.73 | 0.390 | 1.0 |
| D-gluconate | 1.72 | 0.348 | 1.0 |
| a-ketoglutarate | 1.70 | 1.0 | 1.0 |
| succinyl-CoA | 1.69 | 1.0 | 1.0 |
| 4-pyridoxic acid | 1.67 | 0.791 | 1.0 |
| hydroxyproline | 1.67 | 0.044 | 1.0 |
| ADP-D-glucose | 1.65 | 0.988 | 1.0 |
| aconitate | 1.65 | 0.038 | 1.0 |
| FAD | 1.65 | 1.0 | 1.0 |
| acetoacetyl-CoA | 1.65 | 1.0 | 1.0 |
| indole | 1.65 | 0.170 | 1.0 |
| acetyllysine | 1.64 | 0.424 | 1.0 |
| dephospho-CoA | 1.61 | 1.0 | 1.0 |
| CMP | 1.61 | 1.0 | 1.0 |
| tryptophan | 1.61 | 0.026 | 1.0 |
| oxaloacetate | 1.59 | 0.225 | 1.0 |
| D-glucarate | 1.59 | 1.0 | 1.0 |
| D-glucarate | 1.59 | 1.0 | 1.0 |
| phenylalanine | 1.59 | 0.132 | 1.0 |
| GTP | 1.58 | 0.416 | 1.0 |
| methionine sulfoxide | 1.58 | 0.957 | 1.0 |
| valine | 1.56 | 0.287 | 1.0 |
| citraconic acid | 1.56 | 0.102 | 1.0 |
| S-methyl-5'-thioadenosine | 1.56 | 1.0 | 1.0 |
| acetyl-glycine | 1.56 | 1.0 | 1.0 |
| dimethylglycine | 1.56 | 0.125 | 1.0 |
| UDP-glucuronate | 1.56 | 1.0 | 1.0 |
| coenzyme A | 1.56 | 1.0 | 1.0 |
| dATP | 1.54 | 0.476 | 1.0 |
| citrate | 1.54 | 0.056 | 1.0 |
| citrate | 1.54 | 0.057 | 1.0 |
| isocitrate | 1.54 | 0.058 | 1.0 |
| guanosine 5'-diphosphate-3'-diphosphate | 1.53 | 1.0 | 1.0 |
| N-acetyl-L-alanine | 1.53 | 0.113 | 1.0 |
| propionyl-glycine | 1.53 | 0.114 | 1.0 |
| ATP | 1.51 | 1.0 | 1.0 |
| lysine | 1.48 | 0.225 | 1.0 |
| 6-phosphogluconolactone | 1.47 | 1.0 | 1.0 |
| phenyllactic acid | 1.47 | 1.0 | 1.0 |
| arginine | 1.47 | 1.0 | 1.0 |
| UDP-D-glucose | 1.46 | 0.186 | 1.0 |
| alanine | 1.44 | 0.347 | 1.0 |
| sarcosine | 1.44 | 0.351 | 1.0 |
| N-acetylputrescine | 1.44 | 0.339 | 1.0 |
| Octoluse Bisphosphate | 1.43 | 1.0 | 1.0 |
| pantothenate | 1.42 | 1.0 | 1.0 |
| S-adenosyl-L-homocysteine | 1.42 | 1.0 | 1.0 |
| glycerol-3-phosphate | 1.42 | 1.0 | 1.0 |
| UTP | 1.41 | 1.0 | 1.0 |
| 2-hydroxy-glutarate | 1.41 | 1.0 | 1.0 |
| glutamate | 1.41 | 1.0 | 1.0 |
| N-acetylaspartate | 1.40 | 1.0 | 1.0 |
| (iso)leucine | 1.37 | 1.0 | 1.0 |
| tyrosine | 1.37 | 0.977 | 1.0 |
| serine | 1.36 | 0.281 | 1.0 |
| histidine | 1.36 | 1.0 | 1.0 |
| lactate | 1.35 | 0.750 | 1.0 |
| N-acetyl-glutamine | 1.35 | 1.0 | 1.0 |
| hydroxyphenylpyruvate | 1.35 | 1.0 | 1.0 |
| glutathione | 1.33 | 0.743 | 1.0 |
| dCDP | 1.33 | 1.0 | 1.0 |
| proline | 1.32 | 1.0 | 1.0 |
| 2-keto-isovalerate | 1.32 | 1.0 | 1.0 |
| cytosine | 1.32 | 1.0 | 1.0 |
| taurine | 1.32 | 1.0 | 1.0 |
| pyroglutamic acid | 1.31 | 1.0 | 1.0 |
| glucose | 1.31 | 1.0 | 1.0 |
| UDP-N-acetyl-glucosamine | 1.30 | 1.0 | 1.0 |
| NADP+ | 1.30 | 1.0 | 1.0 |
| fumarate | 1.30 | 1.0 | 1.0 |
| methylnicotinamide | 1.29 | 1.0 | 1.0 |
| glycine | 1.29 | 0.646 | 1.0 |
| methionine | 1.28 | 1.0 | 1.0 |
| ophthalmic acid | 1.28 | 1.0 | 1.0 |
| glutamine | 1.27 | 1.0 | 1.0 |
| Methylmalonic acid | 1.27 | 1.0 | 1.0 |
| succinate | 1.27 | 1.0 | 1.0 |
| homoserine | 1.27 | 0.375 | 1.0 |
| threonine | 1.27 | 0.378 | 1.0 |
| cystine | 1.26 | 1.0 | 1.0 |
| NAD+ | 1.25 | 1.0 | 1.0 |
| 3-S-methylthiopropionate | 1.22 | 1.0 | 1.0 |
| creatine phosphate | 1.21 | 0.306 | 1.0 |
| 1-methyl-histidine | 1.21 | 1.0 | 1.0 |
| pyridoxine | 1.21 | 1.0 | 1.0 |
| 6-phosphogluconate | 1.21 | 1.0 | 1.0 |
| malate | 1.20 | 0.151 | 1.0 |
| 1_3-diphosphoglyceric acid | 1.18 | 1.0 | 1.0 |
| 3-phosphoglycerate | 1.18 | 1.0 | 1.0 |
| FMN | 1.18 | 1.0 | 1.0 |
| glycerate | 1.18 | 1.0 | 1.0 |
| Sedoheptoluse bisphosphate | 1.17 | 1.0 | 1.0 |
| creatine | 1.16 | 1.0 | 1.0 |
| D-sedoheptulose-1/7-phosphate | 1.16 | 1.0 | 1.0 |
| sedoheptoluse-7-phosphate | 1.16 | 1.0 | 1.0 |
| NADH | 1.13 | 1.0 | 1.0 |
| N-acetyl-glucosamine-6-phosphate | 1.13 | 1.0 | 1.0 |
| aspartate | 1.13 | 0.518 | 1.0 |
| ribose-5-phosphate | 1.12 | 1.0 | 1.0 |
| pyruvate | 1.11 | 1.0 | 1.0 |
| homocysteine | 1.11 | 1.0 | 1.0 |
| Methylcysteine | 1.11 | 1.0 | 1.0 |
| inosine | 1.10 | 1.0 | 1.0 |
| glucose-6-phosphate | 1.10 | 1.0 | 1.0 |
| xanthosine-5-phosphate | 1.08 | 1.0 | 1.0 |
| D-glyceraldehdye-3-phosphate | 1.08 | 1.0 | 1.0 |
| dihydroxyacetone phosphate | 1.08 | 1.0 | 1.0 |
| fructose-1-6-bisphosphate | 1.07 | 1.0 | 1.0 |
| folate | 1.06 | 1.0 | 1.0 |
| ribulose-5-phosphate | 1.02 | 1.0 | 1.0 |
| creatinine | 0.99 | 1.0 | 1.0 |
| phosphoenolpyruvate | 0.98 | 1.0 | 1.0 |
| glutathione disulfide | 0.93 | 1.0 | 1.0 |
| cytidine | 0.81 | 1.0 | 0.373 |
| aminoadipic acid | 0.68 | 1.0 | 0.122 |
| imidazoleacetic acid | 0.68 | 1.0 | 1.0 |
| hydroxyphenylacetic acid | 0.67 | 1.0 | 1.0 |
| 3-phospho-serine | 0.61 | 1.0 | 0.121 |
| 5-phosphoribosyl-1-pyrophosphate | 0.57 | 1.0 | 0.220 |
| N-carbamoyl-L-aspartate | 0.56 | 1.0 | 0.017 |
